# The Effects of Induction and Treatment of Intracranial Hypertension on Cerebral Autoregulation: An Experimental Study

**DOI:** 10.1155/2018/7053932

**Published:** 2018-06-25

**Authors:** Marcelo de Lima Oliveira, Angela Macedo Salinet, Ricardo de Carvalho Nogueira, Alessandro Rodrigo Belon, Wellingson Silva Paiva, Brasil Chian Ping Jeng, Manoel Jacobsen Teixeira, Edson Bor-Seng-Shu

**Affiliations:** Division of Neurosurgery, Hospital das Clinicas da Faculdade de Medicina da Universidade de Sao Paulo, Sao Paulo, Brazil

## Abstract

**Background:**

This study aimed to analyse cerebral autoregulation (CA) during induction and treatment of intracranial hypertension (ICH) in an experimental model.

**Materials and Methods:**

Landrace and Duroc piglets were divided into mild and severe ICH groups. Four or seven millilitres of saline solution was infused into paediatric bladder catheter inserted in the parietal lobe (balloon inflation). After 1.5 h, a 3% saline solution was infused via venous catheter, and 30 min later, the bladder catheter balloon was deflated (surgery). The cerebral static autoregulation (sCA) index was evaluated using cerebral blood flow velocities (CBFV) obtained with Doppler ultrasound.

**Results:**

Balloon inflation increased ICP in both groups. The severe ICH group showed significantly lower sCA index values (p=0.001, ANOVA) after balloon inflation (ICH induction) and a higher sCA index after saline injection (p=0.02) and after surgery (p=0.04). ICP and the sCA index were inversely correlated (*r*=−0.68 and p<0.05). CPP and the sCA index were directly correlated (*r*=0.74 and p<0.05).

**Conclusion:**

ICH was associated with local balloon expansion, which triggered CA impairment, particularly in the severe ICH group. Moreover, ICP-reducing treatments were associated with improved CA in subjects with severe ICH.

## 1. Introduction

Cerebral autoregulation (CA) is the mechanism that maintains adequate cerebral blood flow (CBF) based on cerebral metabolism independent of fluctuations in systemic arterial blood pressure (ABP). This process is controlled by three main mechanisms: myogenic, metabolic, and neurogenic [1] that function together to provide adequate energy substrates for cerebral metabolic demands and to protect against variations in ABP. When CA is impaired, CBF tends to passively vary with changes in ABP. This condition can lead to cerebral hyperaemia or oligaemia in cases of higher or lower ABP, respectively, and these consequences are linked to brain oedema and intracranial hypertension (ICH). Several previous studies have revealed a relationship between CA failure and both ICH and poor patient outcomes [2–4].

Some factors linked to hyperaemia have been found to trigger ICH because impaired pressure reactivity in cerebral microvessels is associated with higher capillary permeability, resulting in interstitial swelling [3, 5] and/or microvessel dilation and increased CBF volume [6]. On the other hand, oligaemia may cause tissue hypoxia and consequent cellular oedema and ICH. Other authors have demonstrated a relationship between impaired CA and ICH secondary to an obstruction in cerebral venous drainage [7, 8].

However, no strong evidence is available in the literature regarding the influence of ICH on CA. The objective of this study was to analyse CA during induced ICH in a nontraumatic experimental model and to determine how ICH treatment affects CA.

## 2. Materials and Methods

The protocol was approved by the “Research Ethical Committee” at Sao Paulo University Medical School. Two-month-old crossbred Landrace and Duroc piglets weighing approximately 18-20 kg were anaesthetized with propofol 5-10 mg/kg/h (1% Provine®), and fentanyl was used for analgesia (Fentanest®, Cristália) at an initial dose of 5 *μ*g/kg followed by a continuous infusion of 0.08-0.15 mg/kg/min. The animals were intubated with an endotracheal tube and were ventilated at a controlled volume (Fan Dixtal® 5010), with a tidal volume of 10 ml/kg and a fraction of inspired oxygen of 0.40. The invasive monitoring of the mean arterial blood pressure (MABP) was performed using a right femoral artery catheter. End tidal CO_2_ (ETCO_2_), peripheral haemoglobin saturation (SpO_2_), and systemic pH were continuously monitored.

### 2.1. Experimental Model Preparation

**Figure 1:**
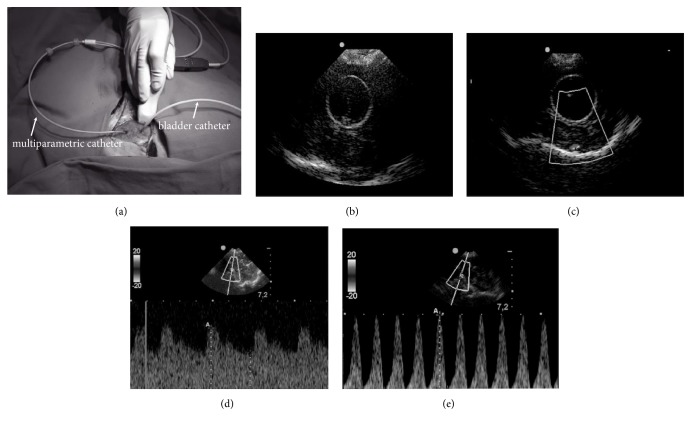
(a) Ultrasound transducer under the lateral hole, a multiparametric catheter in the anterior hole, and a bladder catheter in the posterior hole. (b and c) B-mode ultrasound duplex demonstrating the inflated balloon. Doppler mode was used to obtain CBFV before (d) and after balloon inflation (e).

Two 3-mm holes were made 1 cm lateral to the metopic suture: one for a multiparameter cerebral catheter to measure intracranial pressure, temperature, and tissue oxygen (microsensor-type microchip, Neurovent-PTiO®; Raumedic), which was placed in a hole anterior to the coronal suture and inserted 1.5 cm deep into the frontal lobe, and the other for a paediatric 8-French bladder catheter, which was placed in a hole 1 cm posterior to the coronal suture and inserted 2 cm deep into the parietal lobe. A third small hole was made in the middle anterior fossa to be used as a window for the duplex ultrasound probe (transducer 4-8 Mhz, MicroMaxx® model, SonoSite®, Bothel, WA) [9] (Figure 1).

### 2.2. Intracranial Hypertension Induction

In this nontraumatic model, after each animal was prepared, the paediatric catheter balloon was progressively inflated with 0.9% saline solution over 15 min using continuous pump infusion until 4 ml or 7 ml was infused to trigger either mild ICH or severe ICH, respectively. The 4-ml volume corresponds to an expansive lesion of 72.7 ml in human adults, and the 7-ml volume corresponds to a lesion of 127.3 ml [1]. Mild ICH was defined as an ICP ≤ 25 mmHg, and severe ICH was defined as an ICP > 25 mmHg. After 1.5 h, 3% saline solution (5.3 ml/kg) was infused via the venous catheter. After 30 min, the balloon was deflated, and this manoeuvre was defined as “surgery”. Physiological parameters were monitored for 1 h after the balloon was deflated. At the end of the experiment, the animals were sacrificed by an overdose of propofol (20 mg/kg) and fentanyl (10 mg/kg) followed by 40 ml of a 19.1% potassium chloride solution.

### 2.3. Cerebral Autoregulation

The cerebral static autoregulation (sCA) index was evaluated using CBF velocities (CBFV) obtained using ultrasound Doppler (MicroMaxx® model, SonoSite®, Bothell, WA, USA). The ultrasound operator was blinded to the ICP and balloon volume. The sCA index was tested before and after each of the following experimental steps: ICH induction, 3% saline infusion, and balloon deflation. The MABP was elevated (20 mmHg) by phenylephrine, and the upper limit of the MABP was 120 mmHg. The initial and final MABPs and CBFVs were recorded to calculate cerebral vascular resistance (CVR) as follows: CVR=MABP/CBFV. The static rate of regulation (sROR) or sCA index was calculated as follows: sROR=100(%ΔCVR/%ΔMABP), where ΔCVR is the change in CVR and ΔMABP is the change in MABP [10].

### 2.4. Statistical Methods

A two-way repeated-measures ANOVA was performed to analyse differences in the effects of the intervention (intracranial hypertension, saline solution, and surgery) on selected variables (ICP, CBFV, MABP, sCA, and EtCO_2_) between the two groups (mild and severe ICH). All the statistical analyses were performed using SPSS (version 12.0; SPSS Inc., Chicago, IL). Significance was set a priori at p<0.05. When significant interactions were identified, we applied Scheffé post hoc tests. Pearson's correlation coefficient analysis was performed for continuous variables.

## 3. Results

A total of 28 piglets were studied. Of these, all the data were collected for the 16 piglets that completed the protocol, while 12 piglets were excluded because they experienced circulatory arrest after the balloon was inflated. The demographic and clinical characteristics of the piglets are summarized in Table 1. No significant differences were observed in the baseline ICP, CBFV, and sCA index between the groups.

### 3.1. Intracranial Hypertension

**Figure 2:**
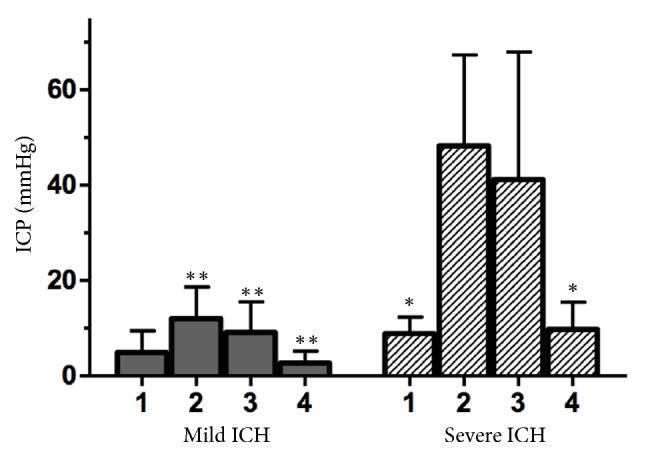
ICP before and after ICH induction and treatment in the mild and severe ICH groups (*∗∗*p<0.03 for the comparison with ICH and *∗*p<0.001 for the comparison with balloon inflation). ICP (intracranial pressure) and ICH (intracranial hypertension); 1 (basal), 2 (balloon inflation), 3 (saline solution infusion), and 4 (balloon deflation).

In response to changes in ICP, ANOVA revealed significant interactions between the groups and between interventions (p=0.01 and p=0.002, respectively). In both groups, balloon inflation resulted in a higher ICP than that recorded at baseline. Moreover, the ICP gradually decreased during the interventions. ICP variations were significant only in the severe ICH group (p=0.007 between baseline and balloon inflation, p=0.04 between baseline and saline solution infusion, and p=0.02 between saline solution infusion and surgery). In the severe ICH group, no significant difference was observed between balloon inflation and saline solution infusion ICP (p=0.87) or between baseline and surgery ICP (p=0.88). ICP was higher in the severe ICH group after balloon inflation and during saline solution injection (p=0.01 for both) (Figure 2, Table 2).

### 3.2. Cerebral Autoregulation

**Figure 3:**
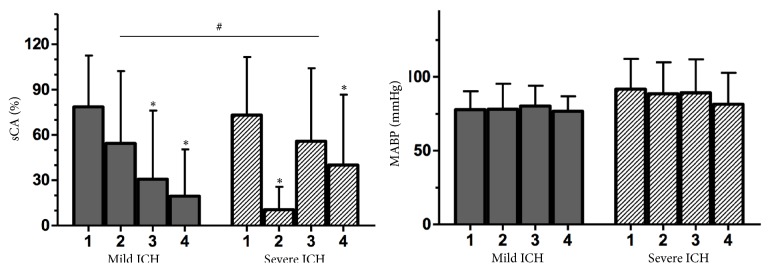
sCA and MABP before and after ICH induction and treatment in the mild and severe ICH groups (#p=0.03 for differences between the groups and *∗*p<0.02 for differences from the first); sCA (static cerebral autoregulation), MABP (mean arterial blood pressure), and ICH (intracranial hypertension); 1 (basal), 2 (balloon inflation), 3 (saline solution infusion), and 4 (balloon deflation).

ANOVA showed that sCA was significantly different between the interventions (p=0.001). Although an increase in ICP led to impaired sCA in both groups, the difference reached statistical significance only in the severe ICH group (p=0.001). In the severe ICH group, an increase in the sCA index was observed after saline was injected (p=0.02) and after surgery (p=0.04) (Figure 3, Table 2). Additionally, in this group, no significant difference in the sCA index was identified between baseline measurements and those obtained after saline solution infusion (p=0.25) or between saline solution infusion and surgery (p=0.44). However, a significant difference was found between the baseline sCA index and the surgery sCA index (p<0.02). In the mild ICH group, a tendency towards a lower sCA index was observed after balloon inflation, but the result was not statistically significant; no improvement in the sCA index was observed after saline solution infusion or surgery (Figure 3, Table 2).

**Figure 4:**
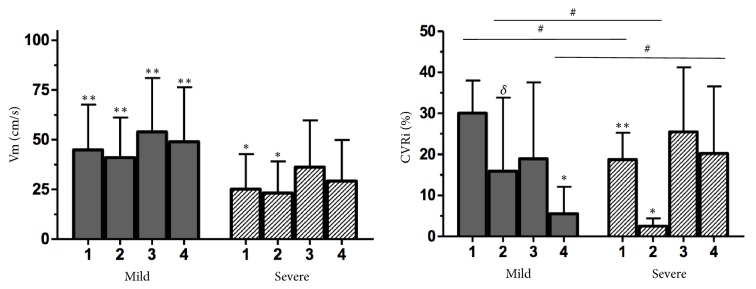
CBFV and CVRi in the mild and severe ICH groups during the experiment; CBFV (*∗∗*p<0.002 for the comparison with ICH and *∗*p=0.04 for the comparison with saline solution) and CVRi (#p<0.02 for differences between the groups, *∗*p<0.02 for differences between the assessments in the same group, *∗∗*p=0.001 for differences between assessments 1 and 3, and §p=0.04 for differences between assessments 1 and 2). CBFV (cerebral blood flow velocity), CVRi (Cerebrovascular resistance index), and ICH (intracranial hypertension); 1 (basal), 2 (balloon inflation), 3 (saline solution infusion), and 4 (balloon deflation).

CBFV was higher in the mild ICH group than that in the severe ICH group (p<0.002). In the severe ICH group, CBFV was higher after saline infusion than that at baseline or after balloon inflation (p=0.04). Additionally, in the severe ICH group, a nonsignificant tendency towards a higher ABP was observed (Figure 4, Table 2).

Statistically significant differences were observed in both groups for CVR after balloon inflation (p=0.04 in the mild ICH group and p<0.02 in the severe ICH group) (Figure 4, Table 2).

**Figure 5:**
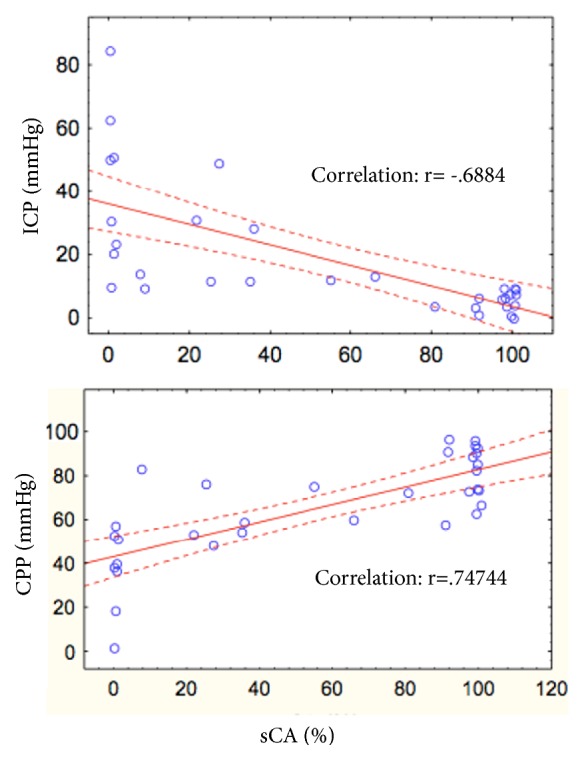
Pearson's correlation analysis of ICP and sCA and of CPP and sCA; ICP (intracranial pressure), CPP (cerebral perfusion pressure), and sCA (static cerebral autoregulation).

Pearson's analysis disclosed an inverse correlation between ICP and the sCA index, showing that a higher ICP was associated with impaired CA (*r*=−0.68 and p<0.05) and a positive correlation between cerebral perfusion pressure (CPP) and the sCA index (*r*=0.74 and p<0.05) (Figure 5).

Concerning systemic pH and ETCO_2_ levels, significant differences were observed between the results at the beginning of the experiment and those obtained at the moment that the balloon was deflated (7.4 and 7.3, respectively (p=0.005), and 37.5 and 38.3 mmHg, respectively (p=0.037)).

## 4. Discussion

The results of the present study clearly indicate CA impairment during ICH and low CPP (Figures 3 and 5, Table 2). One previous study that used the same technique in dogs also found CA impairment during ICH, but conventional methods were not used to study CA, and the effects of ICH relief on CA were not assessed [11]. In contrast, another study applying the same model in rats resulted in intact CA with an ICP between 25 to 30 mmHg [12]. In our study, the sCA index was reduced in the subjects with an ICP≤25 mmHg (the mild ICH group) after balloon inflation, but without statistical significance (Figure 3, Table 2). This research also demonstrated the relationship between ICH and CA impairment: a higher ICP and lower CPP corresponded to a more impaired sCA index (Figure 5).

Some experimental studies have demonstrated that CVR during severe ICH was reduced, possibly to compensate for a decrease in CPP [7, 8, 13–16]. This finding is likely associated with a response delay or a lack of microvascular reactivity during changes in ABP. The present study disclosed a significant decrease in CVR after balloon inflation in both groups. In severe ICH group there was an important CVR increase after infusion of saline solution that remained adequate after balloon deflation; it is important to notice the relation between CVR and sCA index in both groups (Figures 3 and 4). Other studies have described a significant reduction in the CA plateau during severe ICH possibly related to CVR decrease [7, 14, 17, 18]. In addition, a reduction in CVR may trigger an increase in the permeability of the blood brain barrier and a consequential expansion of the free cortical water content [14], which may be an additional factor increasing ICP. However, whether impaired CA triggers ICH or vice versa remains uncertain; a recent meta-analysis demonstrated a strict relationship between CA impairment and ICH but did not answer this question [19]. The results of this study clearly indicate that local balloon inflation, which triggers severe ICH, is associated with CA impairment without involving any toxic and/or inflammatory mechanisms; notably, a tendency towards CA impairment was observed in the subjects with mild ICH despite lack of statistical significance. Many experimental models have been proposed to assess CA during ICH [7, 9, 11–16, 18, 20–31]. Almost all these models involved methods that were associated with impaired CA during normal ICP, such as closed TBI, cerebral haemorrhage [28, 29], and mock CSF [15, 16].

Determining the CBFV of larger intracranial arteries by Doppler was a useful method for estimating CBF in the animals in this study. CBFV was significantly decreased during severe ICH despite increased systemic blood pressure and a subsequent CVR reduction to compensate for the decrease in CPP (Figures 3 and 4, Table 2). Therefore, the association between lower CBFV and CA impairment may lead to severe oligaemia during reduced MABP in subjects with ICH.

During ICH treatment, osmotic agents can improve regional CBF, increase CPP, and decrease ICP [32, 33]; 20% hypertonic saline may reduce CA impairment before ICH relief and CPP elevation in patients with TBI [34, 35]. In the present study, in subjects with severe ICH, 3% hypertonic saline solution more effectively improved CA compared to decreased ICP. This outcome reinforces the hypothesis that osmotic agents can influence impaired CA, likely by promoting an increase in CVR [33]. After 3% osmotic solution was infused in the subjects with mild and severe ICH, CBFV and CVR also increased (Figures 4, Table 2), possibly because of reduced cerebral free water levels and expanded intravascular plasma volume [32–34]. In addition, the decrease in ICP after infusion of hypertonic saline solution resulted in improved intracranial compliance and a consequential increase in CBFV.

After the balloon was deflated, an abrupt reduction in ICP was observed (Figure 2, Table 2). Notably, after the two steps of the study during which severe ICH was reduced, the sCA index improved (Figure 3, Table 2). A sudden reduction of severe ICH may be associated with substantial microvascular dilatation, likely because of evident oligaemia and cerebral lactate elevation during a severe increase in ICP (Figure 4). Some authors have disclosed that NADPH levels are markedly increased during ICH, resulting in cerebral acidosis and impaired CA [14]. Moreover, persistent severe ICH is likely associated with hypoxia and mitochondrial dysfunction, which can enhance cerebral acidosis [36–39]. Therefore, prolonged ICH and cerebral acidosis support cerebral hyperaemia after a decrease in ICP. However, shorter periods of severe ICH followed by ICP relief have been associated with normal CBF [1, 6]. The subjects in the present study were exposed to two hours of mild and severe ICH [9]. We hypothesize that the final sCA index did not return to the baseline sCA index because of cerebral acidosis, systemic acidosis, and elevated ETCO2, which were more significant at the end of the experiment. However, the sCA index during the last step of the study was clearly better than the sCA index after balloon inflation in the severe ICH group (Figure 3). In the mild ICH group, the sCA index was severely impaired associated with decrease in CVR.

The main limitation of our research was the instability of the subjects. Systemic acidosis and higher ETCO_2_ levels after balloon deflation were observed and likely affected the sCA index in both the mild ICH and severe ICH groups. This result was particularly true in the mild ICH group in which CA was completely impaired by the end of the experiment and after infusion of saline solution. The study was performed in immature animals, and the results could potentially be different in adult subjects. Other limitations prevented us from performing a dynamic CA technique, and despite the strength of the correlation between the static and dynamic CA, dynamic data would add important information to our findings.

## 5. Conclusions

The results of the present study indicate that ICH triggers CA impairment, and saline solution and surgery used to relieve high ICP can improve CA in association with ICP reduction.

## Figures and Tables

**Table 1:** Demographic and clinical characteristics of the piglets (SD); ICH (intracranial hypertension), ETCO_2_ (end tidal CO_2_), CBFv (cerebral blood flow velocity), MABP (mean arterial blood pressure), and ICP (intracranial pressure).

****	**mild ICH n=8**	**severe ICH n=8**
gender [%]	F 3 [37.8]	F 5 [62.5]
weight kg	19.12 (0.7)	19.31 (1.0)
ETCO2 mmHg	41 (5.8)	41.62 (4.4)
CBFv cm/s.1	44.8 (22.8)	25.0 (7.5)
MABP mmHg	77 (12.5)	91.6 (7.6)
ICP pre insufflation	4.88 (4.6)	8.87 (3.4)

**Table 2:** Main findings in the different steps of the experiment (SD); *∗*p<0.02 in relation to basal, *⍏*p<0.02 in relation to inflation, *∗∗*p<0.05 for comparison with severe ICH, *✣* p=0.04 in relation to saline, #p=0.01 for differences between assessments basal and saline, *λ* p<0.05 for differences between other assessments between the same group, Ω p=0.04 in relation to basal. sCA (static cerebral autoregulation), ICP (intracranial pressure), CBFV (cerebral blood flow velocity), CVR (cerebral vascular resistance), MABP (mean arterial blood pressure), and ICH (intracranial hypertension).

**mild ICH**					**severe ICH**			
****	**Basal**	**Balloon inflation**	**Saline solution**	**Balloon deflation**	**Basal**	**Balloon inflation**	**Saline solution**	**Balloon deflation**
**sCA index [%]**	78.32 (33.88)	66.73 (96.23)	35.85 (46.59)*∗*	12.60 (30.96)*∗*	74.75 (39.96)	10.56 (15.05)*∗*	57 (49.53)	41.21 (48.36)*∗*
**ICP [mmHg]**	4.88 (4.6)	12.02 (6.64)*∗∗*	9.88 (6.32)*∗∗*	2.7 (2.53)*∗∗*	8.87 (3.45)*⍏*	48.26 (19.05)	41.23 (26.73)	9.78 (5.67)*⍏*
**CBFV [cm/s]**	44.80 (22.8)*∗∗*	41.03 (20)*∗∗*	53.83 (27.14)*∗∗*	48.98 (27.36)*∗∗*	25.06 (7.55)*✣*	23.21 (7.71)*✣*	36.16 (21.52)	29.18 (11.01)
**CVR [%]**	28 (20)	9 (34)Ω	16 (35)*λ*	-5 (21)	18 (10)#	-12 (21)*λ*	20 (36)	10 (42)
**MABP [mmHg]**	77.71 (12.54)	78 (17.26)	80.14 (13.78)	76.71 (10.06)	91.62 (7.68)	88.5 (12.48)	89.12 (18.37)	81.37 (18.81)

## Data Availability

The data used to support the findings of this study are available from the corresponding author upon request.
